# Evaluation of potential of targeted sequencing through mutational signature simulation

**DOI:** 10.1371/journal.pone.0326071

**Published:** 2025-06-25

**Authors:** Keisuke Kodama, Yiwei Ling, Hiroshi Ichikawa, Toshifumi Wakai, Shujiro Okuda

**Affiliations:** 1 Division of Bioinformatics, Niigata University Graduate School of Medical and Dental Sciences, Niigata, Japan; 2 Medical AI Center, Niigata University School of Medicine, Niigata, Japan; 3 Division of Digestive and General Surgery, Niigata University Graduate School of Medical and Dental Sciences, Niigata, Japan; BMSCE: BMS College of Engineering, INDIA

## Abstract

**Background:**

Targeted sequencing is critical in cancer diagnosis, treatment selection, and monitoring. However, the effectiveness of these methods for reflecting whole-exome sequencing (WES)-level mutational signatures remains unclear. Therefore, we addressed this issue through simulation-based analysis to clarify how well targeted sequencing can reproduce WES-level mutational signatures.

**Methods:**

We compared the correlation and similarity of mutational signatures between whole-exome sequencing-level mutation data and downsampled data for gene sets targeted by each sequencing method in 13 cancer types. Additionally, a similarity analysis of the mutational signatures was conducted using randomly downsampled data.

**Results:**

The comparison between whole-exome sequencing and targeted sequencing showed a low correlation based on Pearson’s correlation coefficient but a high similarity based on the Dice similarity index. As a result of the downsampling of data with cancer-related genes and whole genes to evaluate similarity, the cancer-related gene random set showed high similarity when 200–400 genes were selected. However, the whole-genome random set required 2–3 times as many genes as the cancer-related gene random set to show high similarity. Among cancer types, colorectal and lung cancers demonstrated high similarity with fewer downsampled genes, whereas breast and prostate cancers required more downsampled genes to achieve high similarity.

**Conclusion:**

This study demonstrated that current clinically used targeted sequencing methods can reflect whole-exome sequencing-level mutational signatures. This suggests that considering the cancer type and average number of gene mutations in each patient when selecting targeted sequencing methods can lead to more effective treatment choices.

## Introduction

Cancer is a gene-related disease, and driver mutations play critical roles in its development and progression [1,2]. Driver mutations are genetic alterations directly involved in the growth and survival of cancer cells, making them important targets for cancer diagnosis and treatment [3]. Because the key driver genes vary depending on the cancer type, analyzing multiple genes simultaneously according to the cancer type is crucial [4]. Utilizing next-generation sequencing (NGS) technologies, it has become possible to analyze genetic sequences rapidly and cost-effectively. Large-scale genetic analyses using whole-exome sequencing (WES) and whole-genome sequencing (WGS) have enabled us to understand human diseases at the genomic level. Targeted sequencing, which simultaneously examines hundreds of cancer-related genes, is utilized as an efficient method to analyze genetic information at the individual patient level [5,6].

Targeted sequencing plays a crucial role in cancer diagnosis, treatment selection, and monitoring. For instance, the Memorial Sloan-Kettering Cancer Center (MSK-IMPACT) can simultaneously analyze mutations in 468 genes and is FDA-approved in the USA [7]. FoundationOne CDx (Foundation Medicine) covers mutations in 324 genes and has been approved by the FDA in the USA, the EU, and Japan [8]. Additionally, there are other targeted sequencing platforms, such as TruSight Oncology Comprehensive (Illumina, USA) and OncoaccuPanel (NGeneBio, South Korea) [9,10]. While these targeted sequencing methods generally use over 300 genes, small-panel methods such as the Oncomine Focus Assay (Thermo Fisher Scientific, USA) focus on 52 cancer-related genes [5]. Targeted sequencing is also available for liquid biopsy samples, such as FoundationOne Liquid (Foundation Medicine, USA) and Guardant 360 CDx (Guardant Health, USA), providing more flexible options for clinical application [11].

Each targeted sequencing method includes representative driver genes; however, the selection of other cancer-related genes differs among the targeted sequencing. On the other hand, there are few effective molecular-targeted drugs for the resulting mutated genes, leading to a low treatment success rate of only 10–20% [12–14]. As these methods use NGS and the cost of one test is high, selecting the appropriate test from the various targeted sequencing panels available is crucial.

These causative gene mutations in cancer can be present in all cells of the human body and occur throughout a person’s lifetime [15,16]. They result from multiple mutational processes, including minor errors during DNA replication, exposure to mutagens, enzymatic DNA modifications such as methylation, and defects in DNA repair [17,18]. A method called “mutational signatures” exists to estimate the causes of these mutations by classifying the patterns of DNA substitution mutations. This method uses 96 different trinucleotide patterns, considering the adjacent bases on the 5’ and 3’ sides of a single base substitution, and classifies them into existing patterns using techniques, such as non-negative matrix factorisation (NMF), to estimate the causative factors [19]. The COSMIC database categorises these mutational signatures into 30 single-base substitution (SBS) patterns (SBS1-SBS30). For example, SBS4, associated with smoking, is commonly observed in smoking-related cancers such as lung cancer, and SBS7, associated with UV-induced DNA damage, is prominently observed in skin cancer [20,21]. By analyzing these mutational signatures, the underlying causes of cancer can be inferred from patient-specific mutation patterns, which cannot be revealed by single gene mutations alone [22].

Calculation of mutational signatures typically uses information from all gene mutations at the WGS or WES level. However, with respect to targeted sequencing, which focuses on cancer-related genes, it remains unclear whether these mutational signatures can be reliably applied to the estimation of actual causative processes. Therefore, this study aims to determine the optimal number of genes for each cancer type by simulating the relationships between the number of genes and mutational signatures, thereby providing the number of cancer-related genes to be analyzed and the rationale for selecting targeted sequencing methods based on the patient’s disease background.

## Materials and Methods

### Mutation data used in this study

For this analysis, WES mutation data from TCGA for 13 cancer types were used: bladder urothelial carcinoma (BLCA) [23], breast invasive carcinoma (BRCA) [24], colon adenocarcinoma (COAD) [25], head and neck squamous cell carcinoma (HNSC) [26], kidney renal clear cell carcinoma (KIRC) [27], lung adenocarcinoma (LUAD) [28], lung squamous cell carcinoma (LUSC) [29], ovarian serous cystadenocarcinoma (OV) [30], prostate adenocarcinoma (PRAD) [31], skin cutaneous melanoma (SKCM) [32], stomach adenocarcinoma (STAD) [33], oesophageal carcinoma (STES) [34], and uterine corpus endometrial carcinoma (UCEC) [35]. Mutation data for each patient were downloaded from cBioPortal (https://www.cbioportal.org/) and analyzed (S1 Table) [36,37].

### Targeted sequencing methods for comparison

Targeted sequencing methods examined were grouped based on the number of genes included. Oncomine Focus Assay (OFA) [38] was a small-panel method with fewer than 100 genes were used. NCC OncoPanel (NCC) [39] and Oncomine Comprehensive Assay v3 (OCA) [40,41] were categorized using the middle panel method with 100–300 genes. FoundationOne CDx (F1CDx) [8,42], CANCERPLEX (CPX) [43], and MSK-IMPACT (MSK) [44] were categorized as large-panel methods with over 300 genes (Table 1). Additionally, the Cancer Gene Census (downloaded on 06/21/2023), which catalogues cancer-related genes, was used as a reference gene set [45,46].

**Table 1:** List of targeted sequencing methods for comparison.

Targeted sequencing	Number of genes	Developer	Group	Reference
Oncomine Focus Assay (OFA)	52	Thermo Fisher Scientific	Small	38
OncoGuide NCC OncoPanel System (NCC)	124	Sysmex	Middle	39
Oncomine Comprehensive Assay v3 (OCA)	161	Thermo Fisher Scientific	Middle	40, 41
FoundationOne CDx (F1CDx)	324	Foundation Medicine	Large	8, 42
CANCERPLEX (CPX)	435	KEW	Large	43
MSK-IMPACT (MSK)	468	Memorial Sloan Kettering Cancer Center	Large	44

### Random gene sets by downsampling

**Fig 1:**
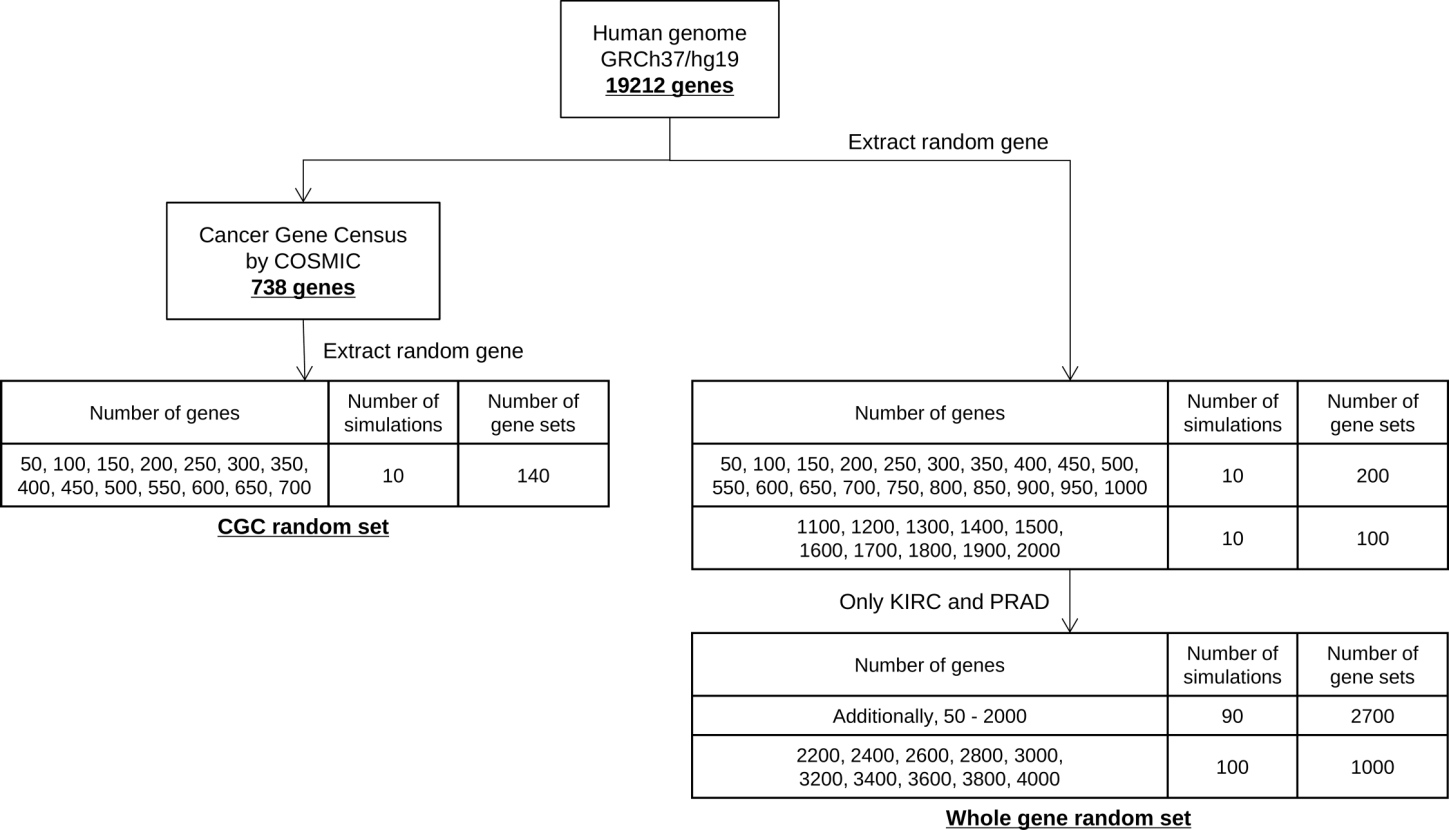
Flowchart for the creation of target gene set.

The Human genome version GRCh37/hg19 was obtained from GENCODE (https://www.gencodegenes.org/human/). As shown in Fig 1, genes were randomly extracted ten times from 19,212 genes encoding exons in the human genome GRCh37/hg19 based on the specified number of genes. These random gene sets were called “Whole gene random set”. Additionally, for KIRC and PRAD only, random gene sets were also created in the range from 2,200–4,000 genes. Moreover, 100 simulation iterations were performed exclusively for KIRC and PRAD. For random sets of cancer-related genes, 738 genes listed in the Cancer Gene Census were randomly extracted 10 times for each specified number of genes (50–700), referred to as the “CGC random set”. A total of 141 gene sets were created using this method. In total, 548 gene sets were used for each of the 11 cancer types, comprising 400 Whole gene random sets, 140 CGC random sets, one CGC gene set, and six targeted sequencing methods (Table 1). For KIRC and PRAD, 4,148 gene sets were used, with an increased number of 4,000 Whole gene random sets while the other components remained the same.

Genes were randomly extracted ten times from 19,212 genes coding for exons in the human genome based on the specified number of genes. The gene lists extracted for each number of genes were used as the CGC random set and the Whole gene random set.

### Mutational signature analysis

DNA substitution data corresponding to the coding genes in the reference gene sets were extracted from WES mutation data for each cancer type. To investigate the relationships between characteristic mutational patterns and cancer causes for each patient, we utilized the mutational signatures from COSMIC (version 2), which identified 30 SBS mutational signatures [16]. The positions and substitution information of SBS variants for each patient were mapped to the COSMIC mutational signatures using the “deconstructSigs” package in R, with the contribution threshold set to less than 0.06. We calculated the mutational signatures for all the 547 gene sets.

### Correlation and similarity analysis

**Fig 2:**
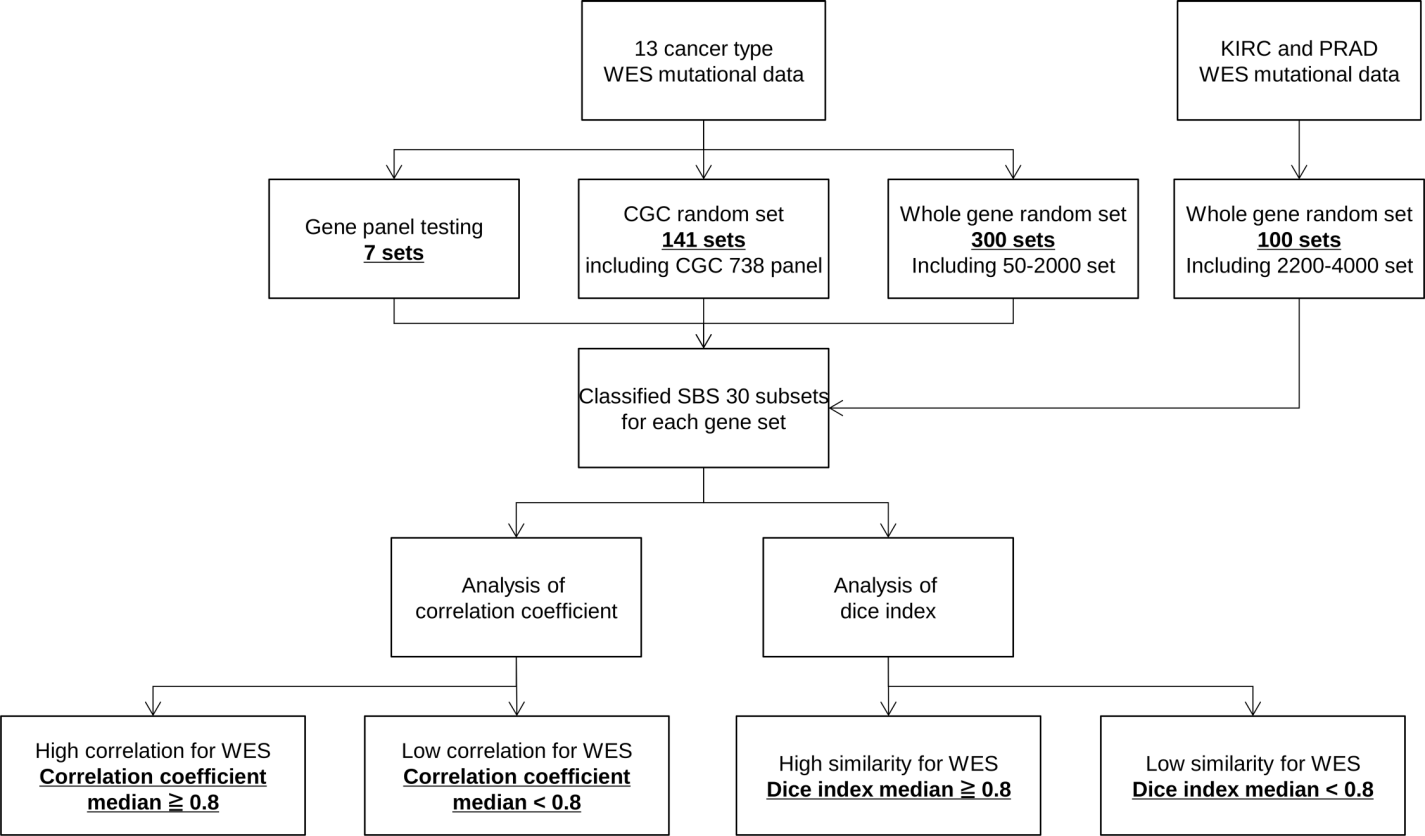
Analysis workflow.


Mutational signatures calculated from mutations in the downsampled gene sets in each patient were compared to those at the WES level before downsampling. Pearson’s correlation analysis and Dice index similarity were used as comparison metrics. The workflow of the analysis is shown in Fig 2. Correlation coefficients were calculated using the following formula:

$$r_{xy}=\frac{\sum_{i=1}^{n}(x_{i}-\overset{―}{x})(y_{i}-\overset{―}{y})}{\sqrt{\sum_{i=1}^{n}(x_{i}-\overset{―}{x}{)}^{2}}\sqrt{\sum_{i=1}^{n}(y_{i}-\overset{―}{y}{)}^{2}}}=\frac{s_{xy}}{s_{x}s_{y}}$$

For Pearson’s correlation analysis, the occurrence frequencies of SBS1 – SBS30 for each patient were calculated as continuous values and used to compare the mutational signatures between the WES mutation data and the downsampled targeted sequencing data. In the calculation of the Dice index, each of the 30 mutational signatures was converted to “1” or “0” if they were present or absent, respectively. These converted data were then compared between a downsampled level and a WES level to calculate similarity. The method for calculating the Dice index was as follows [47]:


$$Dice\;index\;(X,\;Y)=\;\frac{2|X\cap Y|}{\left|X|+\;|Y\right|}$$

To visualize the correlation coefficients and similarities between each gene set, Python (version 3.8.8) Matplotlib was used. Pearson’s correlation analysis and similarity evaluation were based on the median values of correlation coefficients and Dice index, with values of 0.8 or higher as high correlation or similarity and values less than 0.8 as low correlation or similarity.

Using gene mutation data from 13 cancer types, mutational signature analysis was conducted, followed by correlation and similarity analysis of the downsampled mutational signatures.

## Results

### Comparison of mutation counts in each cancer type

**Fig 3:**
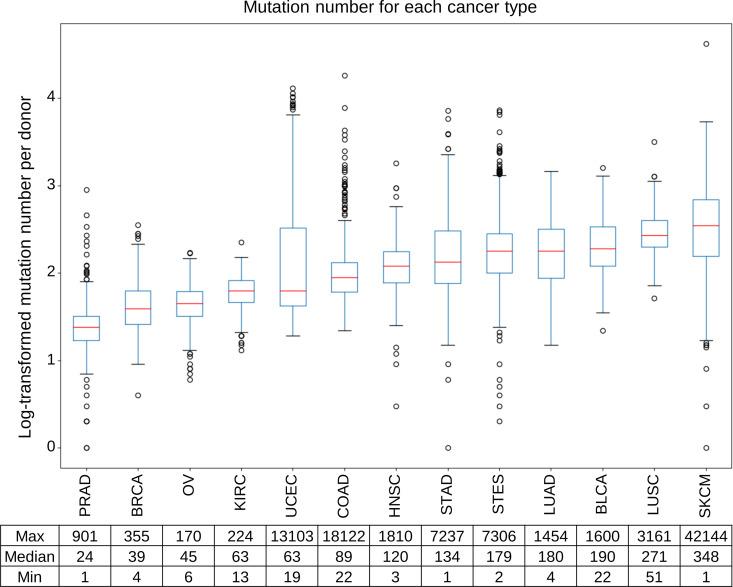
Distribution of gene mutation counts per donor in each cancer type.

The number of single nucleotide variants (SNVs) in the WES data for each patient among the 13 cancer types was compared (Fig 3). The maximum, median, and minimum numbers of somatic mutations for each cancer type are shown in Fig 3. Notably, SKCM, UCEC and COAD included cases with exceptionally high mutation counts exceeding 10,000. SKCM is characterized by a high propensity for mutation accumulation due to repeated DNA damage and repair caused by the exposure of epidermal cells to ultraviolet (UV) radiation [32]. UCEC and COAD are among cancer types with a high frequency of microsatellite instability (MSI), which likely accounts for the presence of cases with elevated mutation burdens [25,35].

The number of gene mutations per donor for each of the 13 cancer types was plotted using boxplots. The number of gene mutations per donor was log-transformed using base 10. The red line indicates the median number of gene mutations in each cancer type. The table shows the maximum, median, and minimum gene mutation counts for donors of each cancer type.

### Correlation and similarity analysis of targeted sequencing

LUAD and COAD, which are known for having a high tumor mutation burden per megabase in coding regions, and BRCA and PRAD, known for having a low tumor mutation burden, were analyzed. For each patient, the correlation and similarity of mutational signatures were calculated using WES-level mutation data and downsampled mutation data based on the gene sets of targeted sequencing.

**Fig 4:**
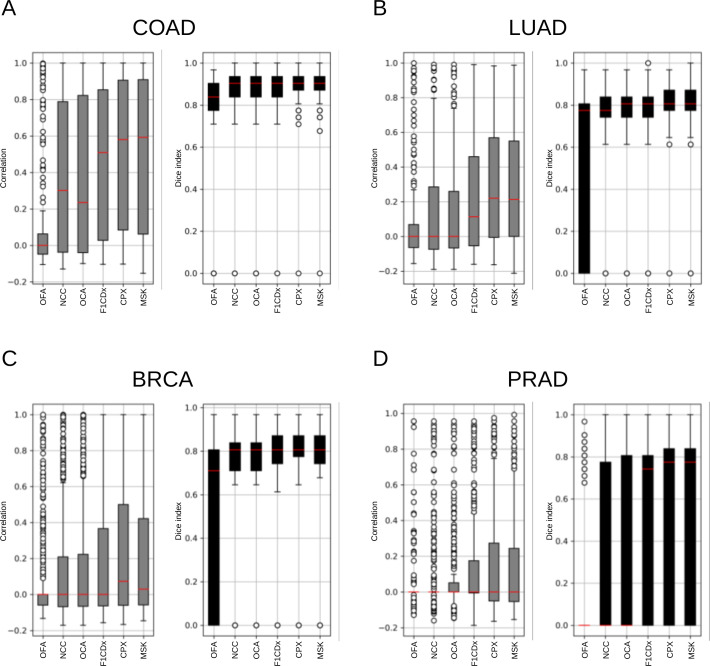
Correlation and similarity of mutational signatures in each targeted sequencing.

Analysis of the correlation between the mutational signatures at the WES level and those downsampled to each targeted sequencing gene set showed that large panel methods had a median correlation coefficient of 0.5–0.6 for COAD, indicating moderate correlation. However, LUAD, BRCA, and PRAD had median correlation coefficients of less than 0.3, making it difficult to find a correlation during downsampling. Therefore, instead of using correlation coefficients to measure the degree of concordance between mutational signature values, similarity was evaluated based on the presence or absence of mutational signatures. High similarity was observed across all three cancer types except PRAD (Fig 4). Specifically, COAD exhibited a high median Dice index close to 0.9, while PRAD showed a median Dice index of 0 in both the small and middle panels, indicating significantly low similarity. Even with the large-panel method, the interquartile range was wide, from 0.0 to 0.8. The same analyzes were conducted for the other nine cancer types, and high similarity with a median Dice index exceeding 0.8, was observed in SKCM, STAD, STES, and BLCA, which have a high tumor mutation burden (S1 Fig).

Boxplots show the correlation coefficients and similarity of mutational signatures before and after downsampling for each donor (A, COAD; B, LUAD; C, BRCA; D, PRAD). The red line indicates median values. The left panel shows the correlation coefficients and the right panel shows the Dice index similarity.

### Correlation and similarity analysis of random gene sets

**Fig 5:**
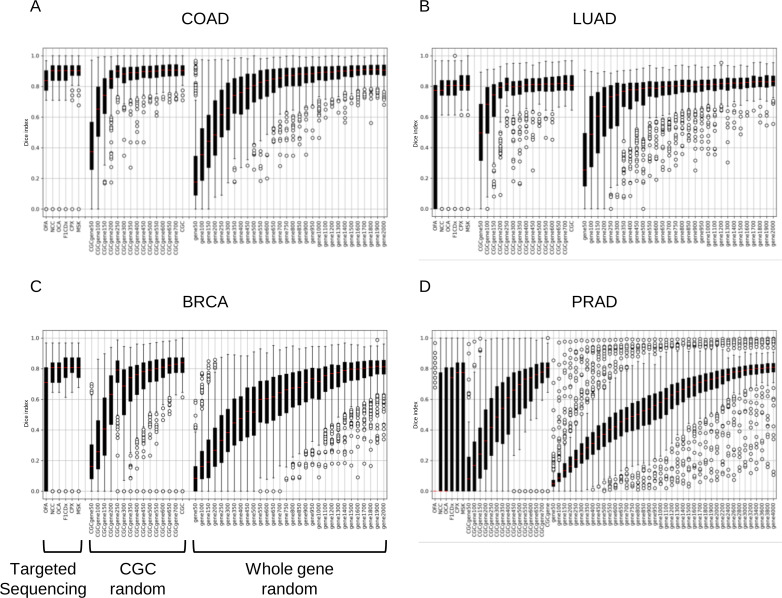
Distribution of mutational signature similarity in each gene set.

Each targeted sequencing method selects important cancer-related genes, including driver genes and other key genes. To verify the significance of the number of genes to be analyzed and the selection of cancer-related genes, the number of genes required to achieve a median Dice index of 0.8 or higher was investigated for the cancer-related gene random set (CGC random set) based on the 738 genes in the Cancer Gene Census (CGC) and the Whole gene random set. As a result of evaluating similarity in the four cancer types (COAD, LUAD, BRCA and PRAD), the CGC random set showed a median Dice index above 0.8 when more than 200 and 300 genes were present in COAD and LUAD, respectively (Fig 5). Conversely, the median Dice index for BRCA was above 0.8 for 600 or more genes, but never exceeded 0.8 for PRAD. When the same analysis was performed on the whole gene random set, COAD and LUAD required 500 and 700 genes, respectively, to exceed the median Dice index of 0.8. Furthermore, BRCA required at least 1800 genes, whereas PRAD did not exceed 0.8, even when 2,000 genes were used.

Boxplots show the similarity of mutational signatures before and after downsampling for each donor (A, COAD; B, LUAD; C, BRCA; D, PRAD). The red line indicates median values. The X-axis represents the gene sets and the Y-axis represents the Dice index. Only PRAD was analyzed for up to 4,000 genes.

### Relationships between the tumor mutation burden and similarity in each cancer type

**Fig 6:**
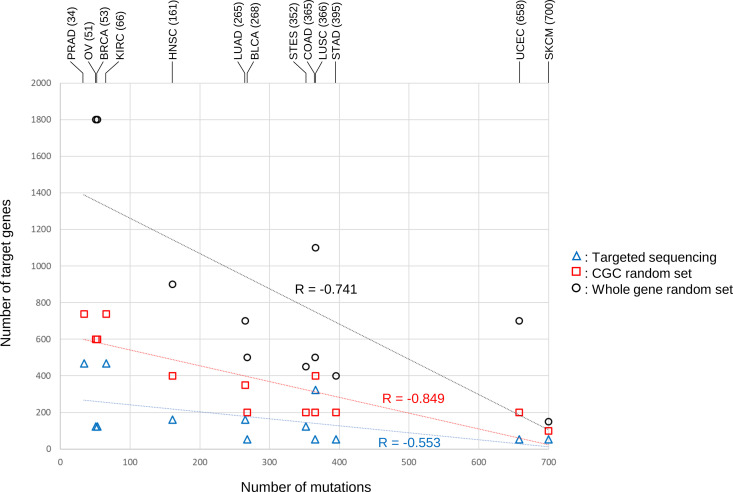
Relationship between the number of gene mutations and the number of genes required to show high similarity in the 13 cancer types.

Cancer types with a higher total number of gene mutations showed higher similarity with fewer target genes after downsampling. Therefore, we visualized the relationship between the number of gene mutations observed in the patients and high similarity (>0.8) (Fig 6). When the number of gene mutations per patient was high, a high similarity was observed, even with fewer target genes. Conversely, cancers with an average number of gene mutations below 100 (PRAD, OV, BRCA, and KIRC) required more than 600 genes to achieve high similarity, even for the CGC random set. In contrast, the Whole gene random set showed high similarity when 2–3 times more genes were used compared to the CGC random set. KIRC and PRAD did not exceed a median Dice index of 0.8 in the 2,000 genes whole gene random set. However, when the Whole gene random set was increased to 4,000 genes, both KIRC and PRAD exceeded 0.8 with 3,200 genes (S2 Fig). The correlation coefficients between the number of genes or mutations and high similarity in the 13 cancer types were calculated for each of the three gene sets. While the CGC random set and Whole gene random set showed a strong negative correlation (r ≦ −0.7), the targeted sequencing showed a moderate negative correlation (r ≈ −0.5).

The results of downsampling in the three types of gene sets showed the number of genes required for each gene set to achieve a median Dice index of over 0.8 (Y-axis). The X-axis represents the average number of gene mutations detected in all donors for each cancer type in the WES data. Cancer types are shown at the top of the graph. Values in parentheses indicate the average number of gene mutations for each cancer type. The number of genes showing high similarity for the Whole gene random set in KIRC and PRAD was 3,200; therefore, these data were excluded from this graph. Moreover, the R-values did not include these two cancer types.

## Discussion

In this study, the correlation and similarity of mutational signatures between the WES-level mutation data and downsampled data for the gene sets of each targeted sequencing method were compared. As a result, the Dice index similarity tended to show higher concordance than the correlation coefficients. This difference arises because Pearson’s correlation assesses how closely the occurrence frequencies of SBS1 – SBS30 align between datasets, whereas the Dice index focuses on whether each signature is present or absent. As the concordance rate between the presence or absence of target mutational signatures is important, a high similarity reflects the characteristics of WES-level mutational signatures. If highly similar results are obtained, this information will be sufficient to help us understand the cause of cancer, as indicated by the mutational signatures provided at the WES level, even with a limited number of genes as in targeted sequencing methods.

Subsequently, to evaluate whether the high similarity depends on cancer-related genes, the similarity was compared between the downsampled cancer-related genes and whole-exon coding genes. Although there were variations among cancer types, the cancer-related gene random set showed notable concordance with the selection of 200–400 genes. In contrast, the whole gene random set did not demonstrate substantial similarity based on the median Dice index unless 2–3 times more genes were selected. This suggests that cancer-related genes play a key role in supporting strong concordance with WES-level mutational signatures. Furthermore, the fact that targeted sequencing achieved comparable performance with even fewer genes than the cancer-related gene random set suggests that targeted sequencing may focus on genes that more efficiently indicate the causes of gene mutations across the genome. Each targeted sequencing method selects many actionable cancer-related genes and important driver genes likely to lead to cancer, which could explain why they reflect mutational signatures better, even with fewer genes. Confirming the presence of mutational signatures in a patient’s cancer DNA could expand treatment options. Therefore, when selecting gene sets for targeted sequencing, incorporating a higher proportion of drivers and other cancer-related genes likely to reflect mutational signatures may lead to effective treatments.

In addition, the number of downsampled genes that showed strong concordance, reflecting WES-level mutational signatures, was investigated for each cancer type. We noted that the total number of gene mutations in each patient varied among cancer types. Focusing on random gene sets, a correlation was found between the number of gene mutations and the number of downsampled genes, showing close resemblance. In other words, a small number of genes in colorectal and lung cancers with high total gene mutation rates can reflect a WES-level mutation signature. In contrast, cancer types with low total gene mutations in each patient, such as breast and prostate cancers, showed high similarity only when a large number of genes were included in the downsampling, suggesting that it is difficult to reflect mutational signatures. For these cancer types, choosing a large-panel targeted sequencing method increases the likelihood of reflecting WES-level mutational signatures. Therefore, selecting targeted sequencing methods that consider the differences in gene mutation load among cancer types would lead to effective treatment proposals based on the causal estimation of cancer using mutational signatures.

Recent studies have reported that mutational signatures inferred from targeted sequencing data are associated with treatment response and prognosis [48]. In addition, systematic CRISPR-based functional screens have demonstrated that targeted disruption of DNA repair genes, such as those involved in mismatch repair or base excision repair, can induce characteristic mutational signatures [49,50]. These findings highlight the biological relevance of mutational signatures and support their potential as functional biomarkers in cancer diagnostics and therapy. However, despite growing interest in the clinical applicability of mutational signatures, no study has systematically and comprehensively evaluated how well targeted sequencing can reproduce WES-level mutational signatures. Our study addresses this gap by simulating downsampling from WES data and quantitatively assessing the preservation of mutational signatures across various gene sets and cancer types. In particular, our findings highlight the potential utility of targeted sequencing for mutational signature analysis in clinical settings where WES or WGS is difficult to implement. Nevertheless, targeted sequencing may not sufficiently recapitulate WES-level mutational signatures in cancer types with a low mutation burden. In addition, this study is based on WES data from TCGA and has limitations including restricted sample size, lack of gene expression information, and the exclusion of non-coding regions. Future research should incorporate larger clinical cohorts and explore the integration of other genomic alterations, such as copy number variations and structural variations, to enable more comprehensive mutational signature profiling. Furthermore, our findings suggest the possibility of capturing cancer-type-specific mutational signatures at the targeted sequencing level, which may in the future provide a foundation for their application in treatment selection or drug prioritization. The accumulation of such knowledge could contribute to the realization of more refined personalized medicine and improved cost-effectiveness in clinical oncology.

## Conclusion

In this study, we demonstrated that the targeted sequencing methods currently used in clinical practice reflect WES-level mutational signatures by downsampling WES-level gene mutation data according to the number of target genes. However, targeted sequencing methods with a large number of genes are more likely to reflect WES-level mutational signatures in cancer types with a low number of gene mutations. Based on these findings, considering the cancer type and average number of gene mutations when selecting a targeted sequencing method for a patient may lead to more effective treatment choices.

## Supporting information

S1 Fig Correlation and similarity of mutational signatures in each targeted sequencing. Box plots showing the correlation coefficients and similarity of mutational signatures before and after downsampling for each donor (A: BLCA, B: HNSC, C: KIRC, D: LUSC, E: OV, F: SKCM, G: STAD, H: STES, I: UCEC). The red line indicates the median values. The left panel shows the correlation coefficients, and the right panel shows the Dice index similarity.
(PPTX)

S2 Fig Distribution of mutational signature similarity in each gene set. Box plots showing the similarity of mutational signatures before and after downsampling for each donor (A: BLCA, B: HNSC, C: KIRC, D: LUSC, E: OV, F: SKCM, G: STAD, H: STES, I: UCEC). The red line indicates the median values. The X-axis represents the gene sets, and the Y-axis represents the Dice index. Only PRAD was analyzed up to 4,000 target genes.
(PPTX)

S1 Table This table shows the data sources, sample sizes, download URLs, raw data, and reference information for 13 cancer types downloaded from cBioPortal. (XLSX)

## References

[1] Stratton MR, Campbell PJ, Futreal PA. The cancer genome. Nature. 2009;458(7239):719–24. doi: 10.1038/nature07943 19360079 PMC2821689

[2] Vogelstein B, Papadopoulos N, Velculescu VE, Zhou S, Diaz LA Jr, Kinzler KW. Cancer genome landscapes. Science. 2013;339(6127):1546–58. doi: 10.1126/science.1235122 23539594 PMC3749880

[3] Garraway LA, Lander ES. Lessons from the cancer genome. Cell. 2013;153(1):17–37. doi: 10.1016/j.cell.2013.03.002 23540688

[4] Kandoth C, McLellan MD, Vandin F, Ye K, Niu B, Lu C, et al. Mutational landscape and significance across 12 major cancer types. Nature. 2013;502(7471):333–9. doi: 10.1038/nature12634 24132290 PMC3927368

[5] Meyerson M, Gabriel S, Getz G. Advances in understanding cancer genomes through second-generation sequencing. Nat Rev Genet. 2010;11(10):685–96. doi: 10.1038/nrg2841 20847746

[6] Kamps R, Brandão RD, Bosch BJ van den, Paulussen ADC, Xanthoulea S, Blok MJ, et al. Next-Generation Sequencing in Oncology: Genetic Diagnosis, Risk Prediction and Cancer Classification. Int J Mol Sci. 2017;18(2):308. doi: 10.3390/ijms18020308 28146134 PMC5343844

[7] Zehir A, Benayed R, Shah RH, Syed A, Middha S, Kim HR, et al. Mutational landscape of metastatic cancer revealed from prospective clinical sequencing of 10,000 patients. Nat Med. 2017;23(6):703–13. doi: 10.1038/nm.4333 28481359 PMC5461196

[8] Frampton GM, Fichtenholtz A, Otto GA, Wang K, Downing SR, He J, et al. Development and validation of a clinical cancer genomic profiling test based on massively parallel DNA sequencing. Nat Biotechnol. 2013;31(11):1023–31. doi: 10.1038/nbt.2696 24142049 PMC5710001

[9] Al-Kateb H, Knight SM, Sivasankaran G, Voss JS, Pitel BA, Blommel JH, et al. Clinical Validation of the TruSight Oncology 500 Assay for the Detection and Reporting of Pan-Cancer Biomarkers. J Mol Diagn. 2025;27(4):292–305. doi: 10.1016/j.jmoldx.2025.01.002 39894076

[10] Kim M, Lee C, Hong J, Kim J, Jeong JY, Park NJ-Y, et al. Validation and Clinical Application of ONCOaccuPanel for Targeted Next-Generation Sequencing of Solid Tumors. Cancer Res Treat. 2023;55(2):429–41. doi: 10.4143/crt.2022.891 36470260 PMC10101781

[11] Maani N, Panabaker K, McCuaig JM, Buckley K, Semotiuk K, Farncombe KM, et al. Incidental findings from cancer next generation sequencing panels. NPJ Genom Med. 2021;6(1):63. doi: 10.1038/s41525-021-00224-6 34282142 PMC8289933

[12] Hilal T, Nakazawa M, Hodskins J, Villano JL, Mathew A, Goel G, et al. Comprehensive genomic profiling in routine clinical practice leads to a low rate of benefit from genotype-directed therapy. BMC Cancer. 2017;17(1):602. doi: 10.1186/s12885-017-3587-8 28854908 PMC5577820

[13] Zhao S, Zhang Z, Zhan J, Zhao X, Chen X, Xiao L, et al. Utility of comprehensive genomic profiling in directing treatment and improving patient outcomes in advanced non-small cell lung cancer. BMC Med. 2021;19(1):223. doi: 10.1186/s12916-021-02089-z 34592968 PMC8485523

[14] Matsubara J, Mukai K, Kondo T, Yoshioka M, Kage H, Oda K, et al. First-Line Genomic Profiling in Previously Untreated Advanced Solid Tumors for Identification of Targeted Therapy Opportunities. JAMA Netw Open. 2023;6(7):e2323336. doi: 10.1001/jamanetworkopen.2023.23336 37459099 PMC10352863

[15] Martincorena I, Campbell PJ. Somatic mutation in cancer and normal cells. Science. 2015;349(6255):1483–9. doi: 10.1126/science.aab4082 26404825

[16] Alexandrov LB, Jones PH, Wedge DC, Sale JE, Campbell PJ, Nik-Zainal S, et al. Clock-like mutational processes in human somatic cells. Nat Genet. 2015;47(12):1402–7. doi: 10.1038/ng.3441 26551669 PMC4783858

[17] Helleday T, Eshtad S, Nik-Zainal S. Mechanisms underlying mutational signatures in human cancers. Nat Rev Genet. 2014;15(9):585–98. doi: 10.1038/nrg3729 24981601 PMC6044419

[18] Alexandrov LB, Kim J, Haradhvala NJ, Huang MN, Tian Ng AW, Wu Y, et al. The repertoire of mutational signatures in human cancer. Nature. 2020;578(7793):94–101. doi: 10.1038/s41586-020-1943-3 32025018 PMC7054213

[19] Alexandrov LB, Nik-Zainal S, Wedge DC, Aparicio SAJR, Behjati S, Biankin AV, et al. Signatures of mutational processes in human cancer. Nature. 2013;500(7463):415–21. doi: 10.1038/nature12477 23945592 PMC3776390

[20] Govindan R, Ding L, Griffith M, Subramanian J, Dees ND, Kanchi KL, et al. Genomic landscape of non-small cell lung cancer in smokers and never-smokers. Cell. 2012;150(6):1121–34. doi: 10.1016/j.cell.2012.08.024 22980976 PMC3656590

[21] Pleasance ED, Cheetham RK, Stephens PJ, McBride DJ, Humphray SJ, Greenman CD, et al. A comprehensive catalogue of somatic mutations from a human cancer genome. Nature. 2010;463(7278):191–6. doi: 10.1038/nature08658 20016485 PMC3145108

[22] Helleday T, Petermann E, Lundin C, Hodgson B, Sharma RA. DNA repair pathways as targets for cancer therapy. Nat Rev Cancer. 2008;8(3):193–204. doi: 10.1038/nrc2342 18256616

[23] Weinstein JN, Akbani R, Broom BM, Wang W, Verhaak RGW, McConkey D, et al. Comprehensive molecular characterization of urothelial bladder carcinoma. Nature. 2014;507(7492):315–322. 10.1038/nature12965 24476821 PMC3962515

[24] Koboldt DC, Fulton RS, McLellan MD, Schmidt H, Kalicki-Veizer J, McMichael JF, et al. Comprehensive molecular portraits of human breast tumours. Nature. 2012;490(7418):61–70. 10.1038/nature11412 23000897 PMC3465532

[25] Muzny DM, Bainbridge MN, Chang K, Dinh HH, Drummond JA, Fowler G, et al. Comprehensive molecular characterization of human colon and rectal cancer. Nature. 2012;487(7407):330–337. 10.1038/nature11252 22810696 PMC3401966

[26] Lawrence MS, Sougnez C, Lichtenstein L, Cibulskis K, Lander E, Gabriel SB, et al. Comprehensive genomic characterization of head and neck squamous cell carcinomas. Nature. 2015;517(7536):576–582. 10.1038/nature14129 25631445 PMC4311405

[27] Creighton CJ, Morgan M, Gunaratne PH, Wheeler DA, Gibbs RA, Robertson G, et al. Comprehensive molecular characterization of clear cell renal cell carcinoma. Nature. 2013;499(7456):43–49. 10.1038/nature12222 23792563 PMC3771322

[28] Collisson EA, Campbell JD, Brooks AN, Berger AH, Lee W, Chmielecki J, et al. Comprehensive molecular profiling of lung adenocarcinoma: The cancer genome atlas research network. Nature. 2014;511(7511):543–550. 10.1038/nature13385 25079552 PMC4231481

[29] Hammerman PS, Voet D, Lawrence MS, Voet D, Jing R, Cibulskis K, et al. Comprehensive genomic characterization of squamous cell lung cancers. Nature. 2012;489(7417):519–525. 10.1038/nature11404 22960745 PMC3466113

[30] Bell D, Berchuck A, Birrer M, Chien J, Cramer DW, Dao F, et al. Integrated genomic analyses of ovarian carcinoma. Nature. 2011;474(7353):609–615. 10.1038/nature10166 21720365 PMC3163504

[31] Abeshouse A, Ahn J, Akbani R, Ally A, Amin S, Andry CD, et al. The molecular taxonomy of primary prostate cancer. Cell. 2015;163(4):1011–1025. 10.1016/j.cell.2015.10.025 26544944 PMC4695400

[32] Akbani R, Akdemir KC, Aksoy BA, Albert M, Ally A, Amin SB, et al. Genomic classification of cutaneous melanoma. Cell. 2015;161(7):1681–1696. 10.1016/j.cell.2015.05.044 26091043 PMC4580370

[33] Bass AJ, Thorsson V, Shmulevich I, Reynolds SM, Miller M, Bernard B, et al. Comprehensive molecular characterization of gastric adenocarcinoma. Nature. 2014;513(7517):202–209. 10.1038/nature13480 25079317 PMC4170219

[34] Kim J, Bowlby R, Mungall AJ, Robertson AG, Odze RD, Cherniack AD, et al. Integrated genomic characterization of oesophageal carcinoma. Nature. 2017;541(7636):169–174. 10.1038/nature20805 28052061 PMC5651175

[35] Getz G, Gabriel SB, Cibulskis K, Lander E, Sivachenko A, Sougnez C, et al. Integrated genomic characterization of endometrial carcinoma. Nature. 2013;497(7447):67–73. 10.1038/nature12113 23636398 PMC3704730

[36] Cerami E, Gao J, Dogrusoz U, Gross BE, Sumer SO, Aksoy BA, et al. The cBio Cancer Genomics Portal: An open platform for exploring multidimensional cancer genomics data. Cancer Discov. 2012;2(5):401–404. https://doi:10.1158/2159-8290.CD-12-0095 22588877 10.1158/2159-8290.CD-12-0095 PMC3956037

[37] Gao J, Aksoy BA, Dogrusoz U, Dresdner G, Gross B, Sumer SO, et al. Integrative analysis of complex cancer genomics and clinical profiles using the cBioPortal. Sci Signal. 2013;6(269):pl1. doi: 10.1126/scisignal.2004088 23550210 PMC4160307

[38] Williams HL, Walsh K, Diamond A, Oniscu A, Deans ZC. Validation of the Oncomine™ focus panel for next-generation sequencing of clinical tumour samples. Virchows Arch. 2018;473(4):489–503. doi: 10.1007/s00428-018-2411-4 30105577 PMC6182325

[39] Sunami K, Ichikawa H, Kubo T, Kato M, Fujiwara Y, Shimomura A, et al. Feasibility and utility of a panel testing for 114 cancer-associated genes in a clinical setting: A hospital-based study. Cancer Sci. 2019;110(4):1480–90. doi: 10.1111/cas.13969 30742731 PMC6447843

[40] Hovelson DH, McDaniel AS, Cani AK, Johnson B, Rhodes K, Williams PD, et al. Development and validation of a scalable next-generation sequencing system for assessing relevant somatic variants in solid tumors. Neoplasia. 2015;17(4):385–99. doi: 10.1016/j.neo.2015.03.004 25925381 PMC4415141

[41] Luthra R, Patel KP, Routbort MJ, Broaddus RR, Yau J, Simien C, et al. A Targeted High-Throughput Next-Generation Sequencing Panel for Clinical Screening of Mutations, Gene Amplifications, and Fusions in Solid Tumors. J Mol Diagn. 2017;19(2):255–64. doi: 10.1016/j.jmoldx.2016.09.011 28017569

[42] Milbury CA, Creeden J, Yip W-K, Smith DL, Pattani V, Maxwell K, et al. Clinical and analytical validation of FoundationOne®CDx, a comprehensive genomic profiling assay for solid tumors. PLoS One. 2022;17(3):e0264138. doi: 10.1371/journal.pone.0264138 35294956 PMC8926248

[43] Eifert C, Pantazi A, Sun R, Xu J, Cingolani P, Heyer J, et al. Clinical application of a cancer genomic profiling assay to guide precision medicine decisions. Per Med. 2017;14(4):309–25. doi: 10.2217/pme-2017-0011 28890729 PMC5580078

[44] Cheng DT, Mitchell TN, Zehir A, Shah RH, Benayed R, Syed A, et al. Memorial Sloan Kettering-Integrated Mutation Profiling of Actionable Cancer Targets (MSK-IMPACT): A Hybridization Capture-Based Next-Generation Sequencing Clinical Assay for Solid Tumor Molecular Oncology. J Mol Diagn. 2015;17(3):251–64. doi: 10.1016/j.jmoldx.2014.12.006 25801821 PMC5808190

[45] Forbes SA, Beare D, Gunasekaran P, Leung K, Bindal N, Boutselakis H, et al. COSMIC: exploring the world’s knowledge of somatic mutations in human cancer. Nucleic Acids Res. 2015;43(Database issue):D805-11. doi: 10.1093/nar/gku1075 25355519 PMC4383913

[46] Sondka Z, Bamford S, Cole CG, Ward SA, Dunham I, Forbes SA. The COSMIC Cancer Gene Census: describing genetic dysfunction across all human cancers. Nat Rev Cancer. 2018;18(11):696–705. doi: 10.1038/s41568-018-0060-1 30293088 PMC6450507

[47] Carass A, Roy S, Gherman A, Reinhold JC, Jesson A, Arbel T, et al. Evaluating White Matter Lesion Segmentations with Refined Sørensen-Dice Analysis. Sci Rep. 2020;10(1):8242. doi: 10.1038/s41598-020-64803-w 32427874 PMC7237671

[48] Yaacov A, Ben Cohen G, Landau J, Hope T, Simon I, Rosenberg S. Cancer mutational signatures identification in clinical assays using neural embedding-based representations. Cell Rep Med. 2024;5(6):101608. doi: 10.1016/j.xcrm.2024.101608 38866015 PMC11228799

[49] Drost J, van Boxtel R, Blokzijl F, Mizutani T, Sasaki N, Sasselli V, et al. Use of CRISPR-modified human stem cell organoids to study the origin of mutational signatures in cancer. Science. 2017;358(6360):234–8. doi: 10.1126/science.aao3130 28912133 PMC6038908

[50] Zou X, Koh GCC, Nanda AS, Degasperi A, Urgo K, Roumeliotis TI, et al. A systematic CRISPR screen defines mutational mechanisms underpinning signatures caused by replication errors and endogenous DNA damage. Nat Cancer. 2021;2(6):643–57. doi: 10.1038/s43018-021-00200-0 34164627 PMC7611045

