# Supplementary material for: Evaluation of potential of targeted sequencing through mutational signature simulation
Source: PLoS One. 2025 Jun 25;20(6):e0326071. doi: 10.1371/journal.pone.0326071 (PMC12193913; doi:10.1371/journal.pone.0326071)
Supplement: S1 Fig — Box plots showing the correlation coefficients and similarity of mutational signatures before and after downsampling for each donor (A: BLCA, B: HNSC, C: KIRC, D: LUSC, E: OV, F: SKCM, G: STAD, H: STES, I: UCEC). The red line indicates the median values. The left panel shows the correlation coefficients, and the right panel shows the Dice index similarity. (PPTX) [file pone.0326071.s001.pptx]

## Slide 1
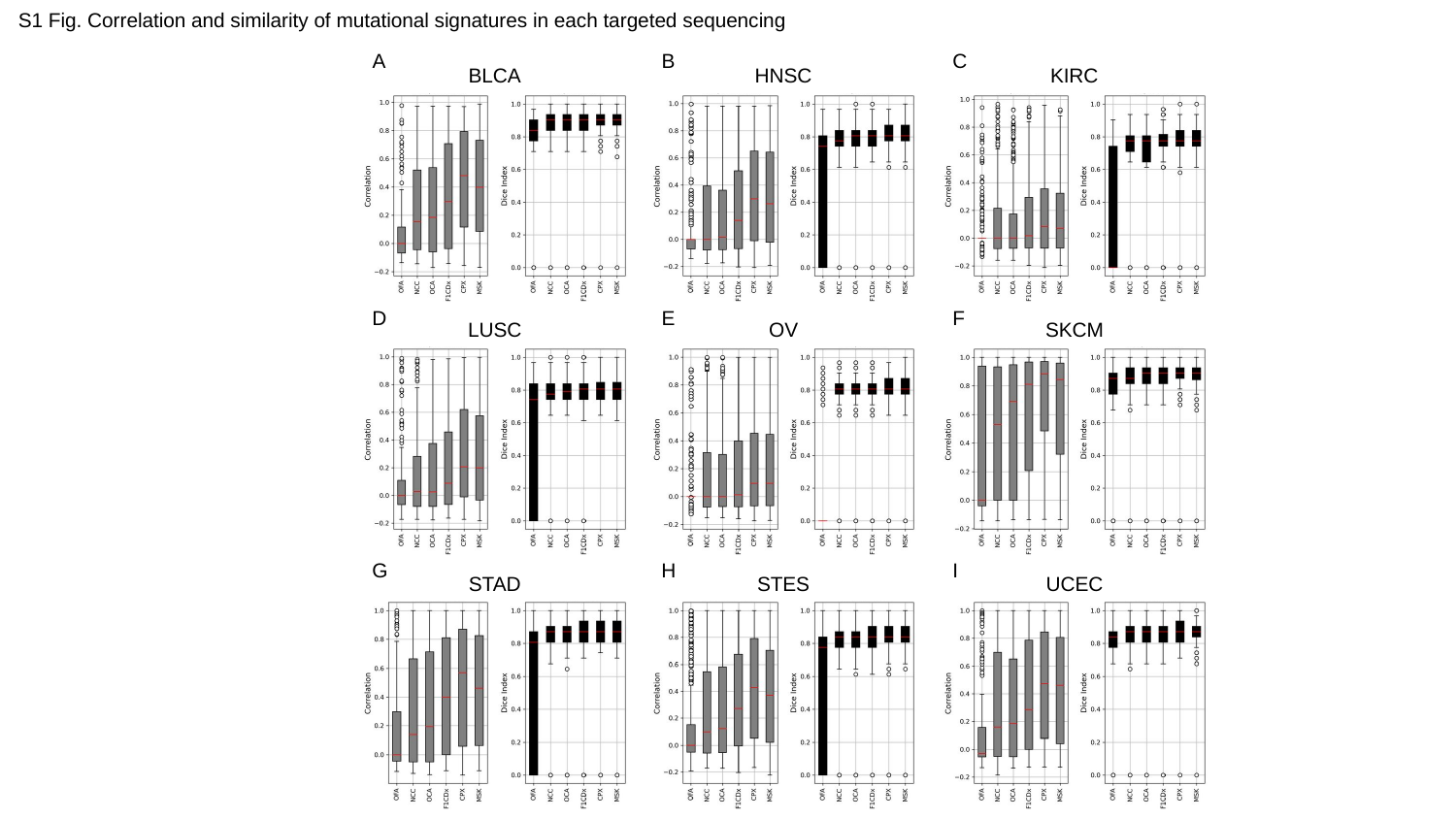

S1 Fig. Correlation and similarity of mutational signatures in each targeted sequencing
A
B
C
BLCA
HNSC
KIRC
D
E
F
LUSC
OV
SKCM
G
H
I
STAD
STES
UCEC
