# Supplementary material for: Evaluation of potential of targeted sequencing through mutational signature simulation
Source: PLoS One. 2025 Jun 25;20(6):e0326071. doi: 10.1371/journal.pone.0326071 (PMC12193913; doi:10.1371/journal.pone.0326071)
Supplement: S2 Fig — Box plots showing the similarity of mutational signatures before and after downsampling for each donor (A: BLCA, B: HNSC, C: KIRC, D: LUSC, E: OV, F: SKCM, G: STAD, H: STES, I: UCEC). The red line indicates the median values. The X-axis represents the gene sets, and the Y-axis represents the Dice index. Only PRAD was analyzed up to 4,000 target genes. (PPTX) [file pone.0326071.s002.pptx]

## Slide 1
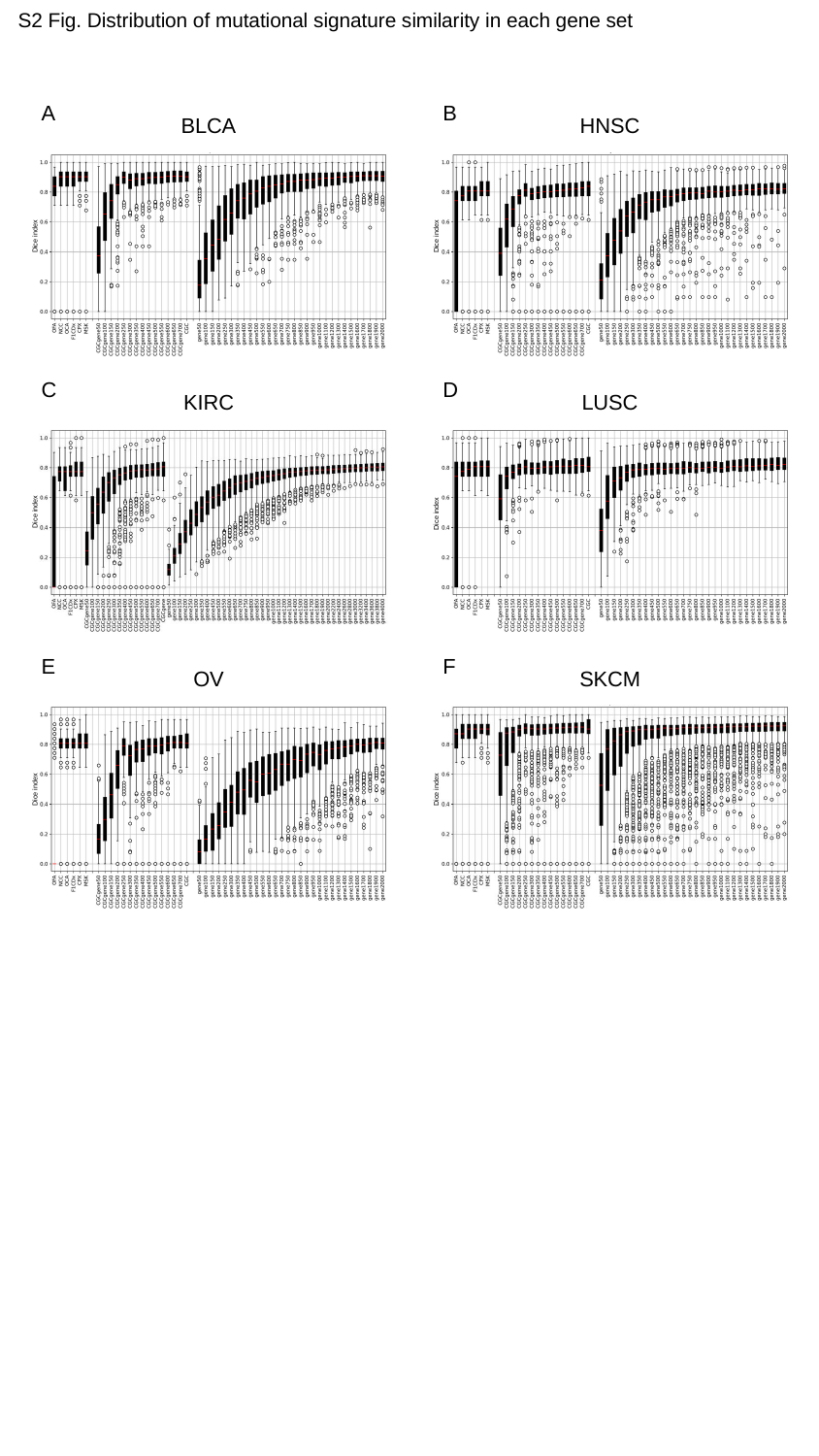

S2 Fig. Distribution of mutational signature similarity in each gene set
A
B
BLCA
HNSC
Dice index
Dice index
Dice index
C
D
KIRC
LUSC
Dice index
Dice index
Dice index
E
F
OV
SKCM
Dice index
Dice index

## Slide 2
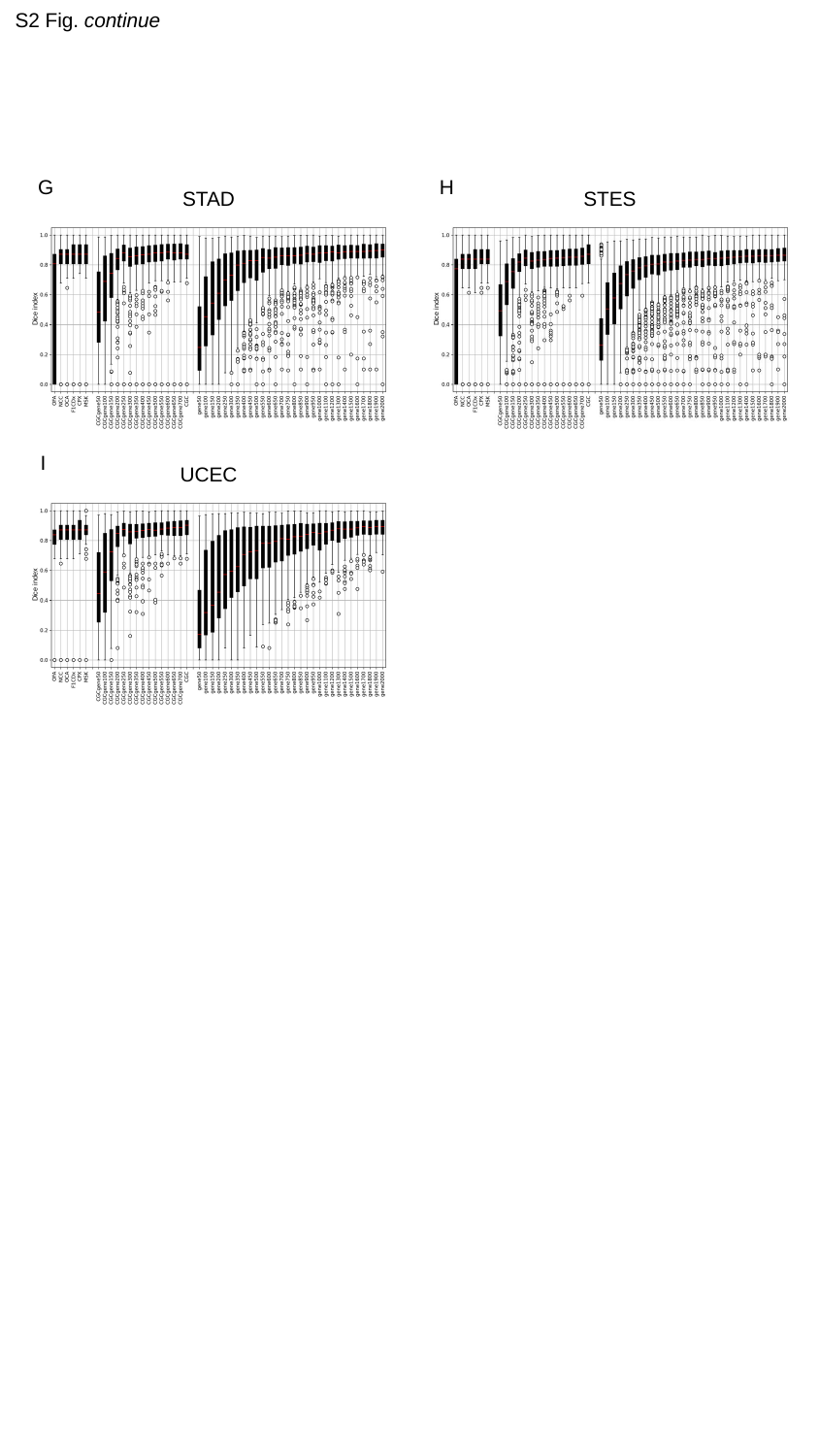

S2 Fig. continue
G
H
STAD
STES
Dice index
Dice index
Dice index
Dice index
Dice index
Dice index
Dice index
Dice index
Dice index
Dice index
I
UCEC
Dice index
Dice index
